# Not Quite Meeting the Mark: College Experiences for Patients With Celiac Disease

**DOI:** 10.1097/PG9.0000000000000392

**Published:** 2023-11-13

**Authors:** Narmeen Khan, Kate Keenan, Hilary Jericho

**Affiliations:** From the * Department of Pediatric Emergency Medicine, The Medical College of Wisconsin Affiliated Hospitals, Children’s Wisonsin, Milwaukee, WI; † Department of Psychiatry and Behavioral Neuroscience, University of Chicago Medicine, Chicago, IL; ‡ Division of Gastroenterology, Hepatology and Nutrition, Celiac Disease Center, Department of Pediatrics, Stanford University, Lucile Packard Children’s Hospital, Palo Alto, CA.

**Keywords:** celiac disease, gluten-free diet, adherence, mental health, adolescence, college

## Abstract

Strict adherence to a gluten-free diet (GFD), the current treatment for celiac disease (CD), is socially challenging for adolescents, especially in the college setting. We conducted a survey of factors contributing to GFD adherence during college among patients meeting the ESPGHAN criteria for CD. One-hundred-one young adults (18 years and older) were contacted; 59 completed the survey, of which 47 were enrolled or had attended college. The survey was developed by the study team. Most patients were able to maintain strict adherence to the GFD, whereas at college and reported that GF food was available and consistent with expectations. Nearly all participants reported a lack of resources for students with CD. Strong family support helped, and school stress and lack of peer support impeded diet adherence. Although colleges may meet the basic needs of celiac students, the availability and quality of gluten-free options, and improved campus resources are needed.

**What Is Known**

- The only current treatment for patients diagnosed with celiac disease (CD) is strict adherence to a gluten-free diet (GFD).
- Strict adherence to a GFD poses many social challenges.
- Adolescence is a time of greatest risk for lack of adherence to the GFD creating the potential for negative medical outcomes.

**What Is New**

- Continued adherence to the GFD is relatively high among college students.
- In general, colleges meet minimum requirements for GF options on campus.
- Significant improvements are needed for greater availability and diversity of GF options on campus as well as support resources for students with chronic medical conditions.

## INTRODUCTION

Chronic illness impacts the social development of adolescents leading to overall lower quality of life and higher rates of depression. College years are a particularly vulnerable time for adolescents with chronic illnesses due to increased concerns surrounding the social impact of their disease and less parental oversight.

Celiac disease (CD) is a chronic, autoimmune disease triggered by the ingestion of gluten (the major storage protein in wheat, barley, and rye) in genetically predisposed individuals, resulting in small intestine inflammation and a wide range of gastrointestinal and extraintestinal manifestations (1). Currently, a gluten-free diet (GFD) is the only treatment for CD. Adherence to a GFD can be a challenge for college students working to intersect food choices with their social life, particularly when options in campus dining halls are limited. Lack of adherence to the GFD, though, can lead to worsening symptom severity, deterioration of the overall well-being of the patient and decreased quality of life (2). Being able to target situations that may be high risk for dietary noncompliance, such as the transition to college or lack of support resources, would be beneficial to help improve rates of dietary compliance during these vulnerable times. Unfortunately, there is little literature currently examining the college experience for patients with CD and its impact on adherence to the GFD.

I purpose of our cross-sectional study was to describe the availability and diversity of gluten-free foods, campus support resources, and adherence to a GFD during the college experience of students with CD.

## METHODS

Participants for this study were initially recruited as part of a study on the emotional health of CD patients. For the initial study, caregivers of consecutive referrals to the University of Chicago pediatric GI clinic who met the ESPGHAN criteria for the diagnosis of CD were asked to consent to collect survey data; youth were asked to assent. Details regarding the initial study recruitment and results have been published (3). Patients who were enrolled in the initial study and who were 18 years and older (n = 101) were contacted via email, text, and/or phone call to participate in a follow-up survey about adjusting to CD in early adulthood. Study participants signed an approved informed consent form before completing the survey (Supplemental Digital Content Figure 1, http://links.lww.com/PG9/A150). After completion of the survey, participants received a $25 gift card for their time. Survey questions and response formats were developed by the authors and surrounded the themes of living and dining conditions at college, availability of gluten-free options, resources available to students, and factors that helped or hindered adherence to the GFD. Answer choices were presented on a Likert scale or as free text. The University of Chicago Institutional Review Board approved all study procedures. The goal of the present study was to provide descriptive data for this population.

## RESULTS

Of the 101 participants contacted, 59 (58%) completed the follow-up survey. Forty-seven of the 59 participants who responded were currently attending or had graduated from college. The study was focused on this subset of respondents. Demographics were available for 46 of the 47 participants. Participants were young adults, ages 18 to 26 years (M = 21.5, SD = 2). Thirty-two patients (70%) identified as female. Living conditions, dining conditions, availability of GF options, support resources, factors that improved adherence to the GFD and factors that worsened adherence to the GFD were assessed in this survey.

### Living Conditions at College

Of the 47 study participants, 20 (42%) of respondents were living on campus in either a dormitory, suite, or fraternity/sorority. The remainder (58%) lived in an off-campus home/apartment, with their parents, or some other living arrangement.

Of the 20 participants who lived in campus housing, 8 (40%) of respondents had access to a private kitchen and 7 (88%) of these respondents found this helpful for adherence to the GFD.

### Dining Conditions at College

**FIGURE 1.**
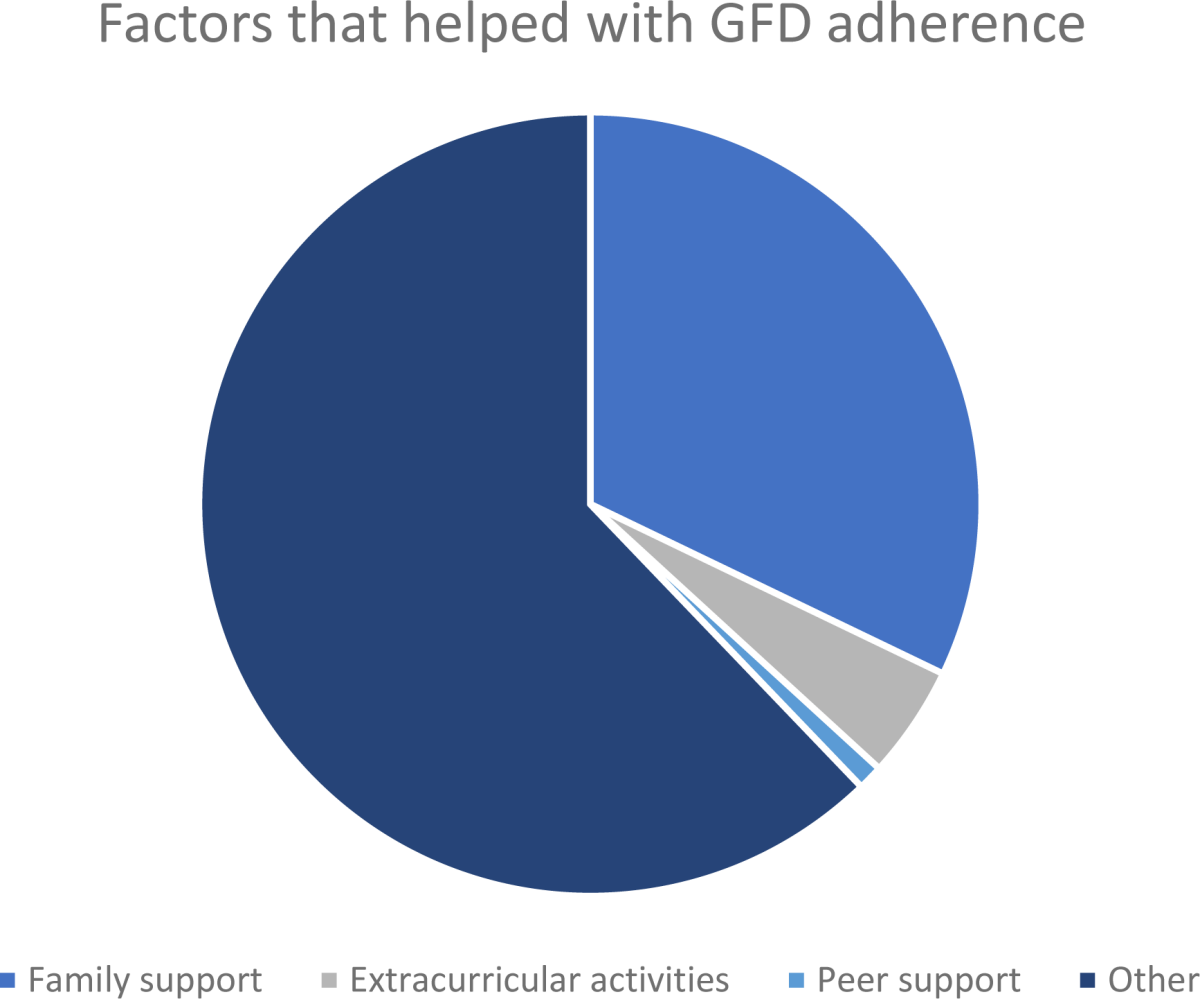
Factors that helped college students with celiac disease adhere to a strict gluten-free diet (GFD).

Of the 20 participants who lived in campus housing, 55% reported that they were offered individually served GF meals, and 45% were offered buffet-style GF meals. Sixty percent of respondents reported that they could almost always find a GF option, 80% reported that the options met their expectations and 62% stated expectations were consistent with the original description provided by the school (Fig. 1). Descriptions of dining experience included general positive and negative comments, concerns about cross-contamination, and the social experience of the dining hall:

#### Positive Comments

“There is always food in the kitchen available for me to choose from or I can place an order for something specific.”

“I enjoyed that the chefs prepare each allergen or celiac student their own meal and place it in a labeled hot box.”

#### Negative Comments

1. Lack of diversity:

“The quality of the food when I was visiting versus when I started attending was very different. I was told I could order anything I want, but when I did, the quality of the food was poor, or they could not accommodate what I originally asked for.”

“On the weekends there are not gluten-free options because the main cafeteria with gluten-free options is closed and the options in the sandwich shops on campus are minimal.”

“There simply is not enough variety. I was told they would be able to cook me things whenever I would like, in reality it was pretty terrible cooking.”

“Some days, there would be very few protein options. I do not eat meat, so those days are much more difficult. I also find that I eat a lot of rice (which is not a bad thing, but I wish there was more variety).”

2. Concerns about cross-contamination:

“I was told the staff was extremely careful about labeling and cross contamination, when in fact they were not.”

3. Social experience of the dining hall:

“I did not like going to the halls with buffets because I had to awkwardly cut the line to ask for them to make gluten-free because it wasn’t ready like the regular food. I would have to wait 15 minutes before I got my food and by that time my friends were done eating.”

### Support Resources on Campus

Only 17% of respondents reported adequate support resources available to them on campus. Of those that did receive a support resource 88% did think that this helped them with adherence to the GFD. The most commonly cited helpful resources included a disability resource center, being provided with extra time on exams and assignments if needed, access to dieticians to review dining options, connections with campus doctors and administration, and the ability to not only create small groups for individuals with dietary restrictions, but also being given access to meet directly with the campus meal service leadership to improve awareness, training and handling of meals for those with special needs. Knowing and being connected with others on campus with similar special dietary needs was not felt to help with adherence to the GFD.

### Factors That Helped Adherence to the GFD at College

Through free text responses, participants reported that factors that helped with adherence to the GFD included family support (62%), fear of worsening symptoms when exposed to gluten (22%), extracurricular activities (9%), and peer support (2%).

### Factors That Worsened Adherence to the GFD at College

**FIGURE 2.**
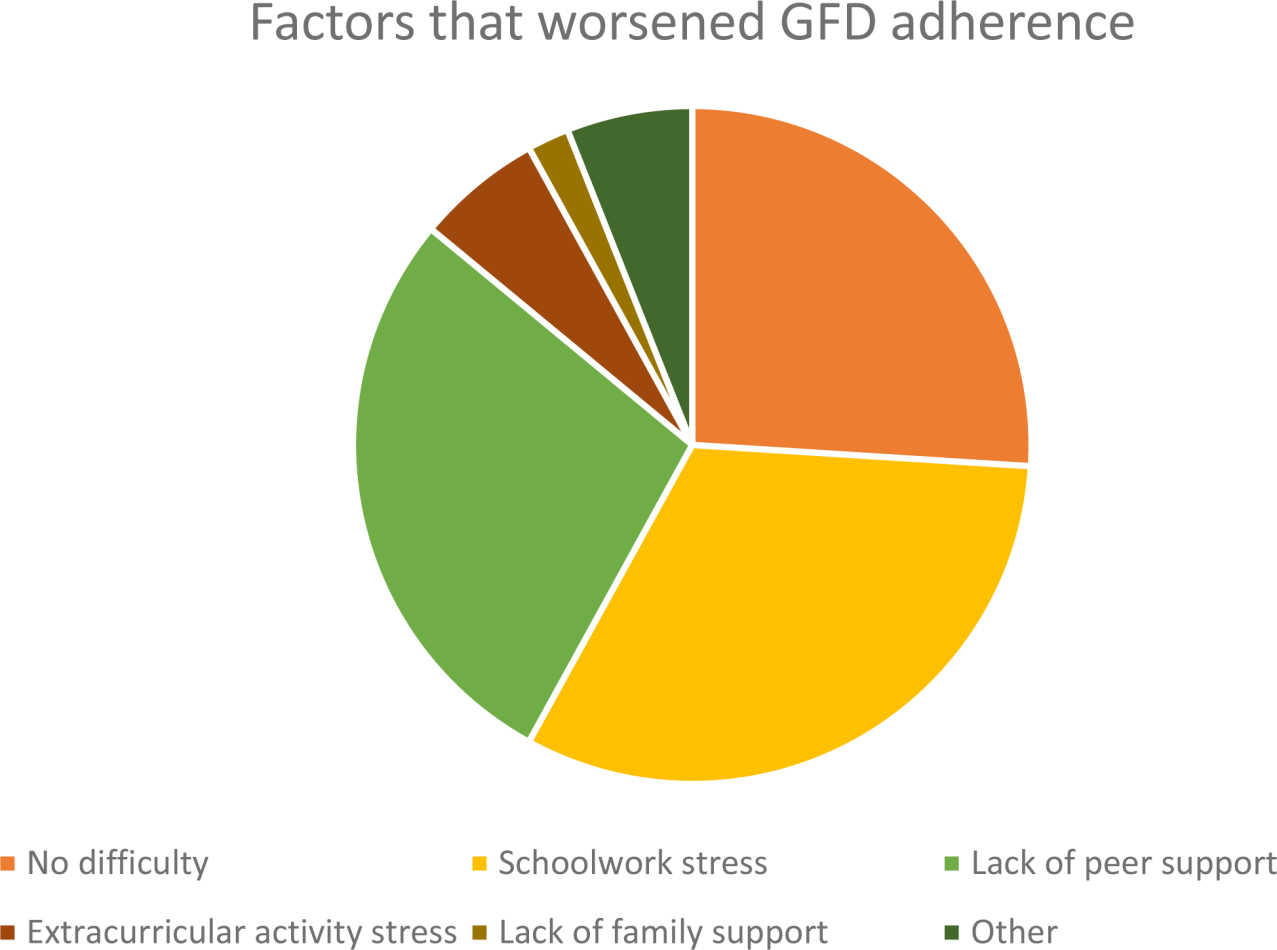
Factors that hindered college students with celiac disease from adherence to a strict gluten-free diet (GFD).

Twenty-six percent of respondents stated that they never had difficulty adhering to the diet. Among those that did report difficulty, factors that worsened adherence to the GFD included stress of schoolwork (32%), lack of peer support (28%), stress from extracurricular activities (6%), and lack of family support (2%). The remaining 6%, independently, reported that a lack of “good” GF options on campus led to a lack of adherence to the GFD during college (Fig. 2).

## DISCUSSION

The main finding of our descriptive study is the identification that the transition to college can be challenging for students with CD. Major support networks available, whereas living at home may be lost when these students transition to college leading to difficulties with sustained adherence to the GFD. The GFD continues to be the only current treatment for CD, but this can cause a significant negative impact on students’ social lives and requires coping strategies while learning to manage their disease independently.

Students with ongoing family support reported the greatest rates of success in ongoing strict adherence to the diet. Schoolwork stress, a lack of peer and family support, extracurricular-related stress, and a lack of adequate, or available, GF options led to the poorest rates of adherence.

The Americans with Disabilities Act (ADA) prohibits discrimination on the basis of disability. To be protected by the ADA, one must have a disability, or have a relationship or association with an individual with a disability. An individual with a disability is defined as a person who has a physical or mental impairment that substantially limits one or more major life activities, a person who has a history of such an impairment, or a person who is perceived by others as having such an impairment. Specifically, public and private accommodations and education “must comply with basic nondiscrimination requirements that prohibit exclusion, segregation and unequal treatment” (4).

In general, colleges do appear to be adhering to the ADA meeting the minimum standards, and hitting the basic needs for students with CD in campus dining halls, but it is clear that greater availability, variety, and diversity of GF options are imperative for maintenance of adherence to the GFD.

Disturbingly, only 17% of respondents reported adequate support resources available to them on campuses. Of those who received support resources, though, 88% felt that this improved adherence to the diet, greatly emphasizing the need. Extra time for exams and assignments, if needed, and access to campus doctors, administration and meal service leadership significantly improved the college experience for students with CD and subsequently led to greater rates of adherence to the GFD.

National organizations, such as Beyond Celiac, offer information for prospective college students with CD. Recommendations include reaching out to the school’s food service director, before arrival, and posing questions such as “Where is the best place to go for gluten-free food? Do any of the dining halls have a gluten-free section for students? Are there other students eating gluten-free on campus? Are you able to share the ingredients of the dishes served so I can ensure they are gluten-free? Do the kitchens have a dedicated section for gluten-free food preparation? How do you prevent cross-contact in your kitchen? Do you use any symbols to indicate which dishes are gluten-free or can be made gluten-free?” Once attending school, students with CD should tell dining room staff that they have a medical condition that requires them to eat gluten-free food and to ask questions before eating including: “This sauce looks great—Can you tell me about the ingredients in it? Do you know if these French fries were cooked with onion rings or chicken fingers? Or are they made in a separate fryer? It looks like some pasta fell into the brown rice. Would you be able to serve me a spoonful of rice from a fresh batch? Do you mind changing your gloves before you prepare my gluten-free sandwich? Can you place my burger on a piece of aluminum foil before you put it on the grill top? I know you toast the buns on the grill, so this way my food will be protected” (5).

Our study highlights that although colleges may have GF options available to students with CD, there may be inexperience and a lack of understanding with respect to the needs of celiac students. Both the quantitative data and the voices of the participants expressed through their thoughtful responses to open-ended questions, provide new insight to families and colleges on ways to better optimize the college experience for this subset of students.

Our study also emphasizes the importance of student support resources on campuses. Colleges that provided support resources to celiac students and created an environment where students could freely interact with administration, food services, and local physicians clearly led to the best student experiences and subsequently the greatest rates of adherence to the GFD. Our findings demonstrate the necessity for greater training to colleges, with regard to the needs of celiac patients, to ensure the smoothest transition during this vulnerable time in patients’ lives.

## LIMITATIONS AND FUTURE DIRECTIONS

Study limitations include the small sample size and the lack of a control group, both of which precluded hypothesis testing. Our multiple methods and attempts to contact the original participants, who were initially enrolled via parent consent and child assent, as consenting adults yielded a retention rate of 58%; a relatively strong rate for this demographic (6,7). The small sample size and use of a nonstandardized or validated survey clearly limit the conclusions that can be drawn from this study. Research with larger, diverse participants using survey methods that have been validated via qualitative and quantitative methods are needed to generate data that can be leveraged to inform college administrations on how to best support their students with CD. In addition, drawing on research with other college students who must comply with dietary restrictions, such as students with food allergies or type 2 diabetes, may be useful in identifying innovations and impediments to supporting the health of a diverse student body.

## CONCLUSION

In conclusion, using this cross-sectional survey study we identified factors described as impacting CD patients’ college experience, related challenges, and factors used to help maintain adherence to the GFD. Our findings offer guidance for improving CD students’ college success, quality of care, and ability to maintain adherence to the GFD during this vulnerable time. Future studies are needed to incorporate a larger sample of CD patients to increase power as well as a comparison groups of college students without CD to allow for causal inference when identifying factors affecting adolescent GFD adherence.

## Supplementary Material



## References

[1] Husby S Koletzko S Korponay-Szabo IR; ESPGHAN Working Group on Coeliac Disease Diagnosis. European society for pediatric gastroenterology, hepatology, and nutrition guidelines for the diagnosis of coeliac disease. J Pediatr Gastroenterol Nutr. 2012;54:136–160. 22197856 10.1097/MPG.0b013e31821a23d0

[2] Mager DR Marcon M Brill H. Adherence to the gluten-free diet and health-related quality of life in an ethnically diverse pediatric population with celiac disease. J Pediatr Gastroenterol Nutr. 2018;66:941–948. 29287009 10.1097/MPG.0000000000001873

[3] Jericho H Khan N Cordova J. Call for action: high rates of depression in the pediatric celiac disease population impacts quality of life. JPGN Rep. 2021;2:e074. 37205970 10.1097/PG9.0000000000000074 PMC10191553

[4] Available at: https://www.ada.gov/resources/disability-rights-guide/#americans-with-disabilities-act-ada.

[5] Available at: https://www.beyondceliac.org/living-with-celiac-disease/school/info-for-college-students.

[6] Francis JKR de Roche AM Mauro C. Adolescent-parent dyadic retention in an interview study and changes in willingness to participate in a hypothetical microbicide safety study. J Pediatr Adolesc Gynecol. 2018;31:592–596. 29906513 10.1016/j.jpag.2018.06.001 PMC6218291

[7] Parrish DE Duron JF Oxhandler HK. Adolescent recruitment strategies: lessons learned from a university-based study of social anxiety. Soc Work Res. 2017;41:213–220.

