# Supplementary material for: Not Quite Meeting the Mark: College Experiences for Patients With Celiac Disease
Source: JPGN Rep. 2023 Nov 13;4(4):e392. doi: 10.1097/PG9.0000000000000392 (PMC10684230; doi:10.1097/PG9.0000000000000392)
Supplement: Supplementary file 1 [file pg9-4-e392-s001.pdf]

Follow Up Survey

Please complete the survey below.

Thank you!

What is your name? (optional)	__________________________________
What is your current living situation?	◯ Living with my parents/caregivers<br>◯ Not living with my parents/caregivers
Which of the following most accurately describes your current situaition?	◯ Working full or part time<br>◯ Attending school<br>◯ Working and attending school<br>◯ Neither working or attending school

College/Dining Environment

Are you attending/have you attended college on-campus (including community college, junior college, or university)?	◯ No<br>◯ Yes, currently attending<br>◯ Yes, graduated or no longer attending
Please describe your living situation when in college.	◯ Shared dormitory<br>◯ Suite<br>◯ Fraternity/Sorority House<br>◯ Off-campus apartment or house<br>◯ I lived at my parent's house<br>◯ Other<br>(If you lived in more than one of these, please choose the one you lived in for the longest.)
Other living situation	__________________________________
Did you have access to a kitchen?	◯ Yes<br>◯ No
Did having access to a kitchen help or hinder you to maintin the gluten free diet	◯ Help<br>◯ Hinder
Why?	__________________________________
What type of on-campus dining services did you have available to you?	◯ Buffet Style<br>◯ Restaurant/Cafeteria style (individually served meals)<br>◯ Other
Other on-campus dining	__________________________________
How often did you feel that the on-campus dining services had gluten-free options?	◯ Never<br>◯ Sometimes<br>◯ Often<br>◯ Almost Always

Please share with us what you liked and disliked about the campus provided gluten-free food options	__________________________________
Did you feel that the gluten-free food available on campus was consistent with the descriptions told to you when initially considering the college?	◯ Yes<br>◯ No
Why not?	______________________________
How often were you able to check the ingredients of the food items of on-campus dining?	◯ Never<br>◯ Sometimes<br>◯ Often<br>◯ Almost Always

Resource Availability

Were you provided with resources (eg: support groups, etc.) regarding celiac disease (or chronic illness in general) while you were in college?	◯ Yes<br>◯ No
What kind of resources were they?	______________________________
Did you feel like these resource(s) were helpful in keeping up with health and/or maintaining a gluten-free diet?	◯ Yes<br>◯ No
Why not?	______________________________
Did you know other students with celiac disease or other chronic illness?	◯ Yes<br>◯ No
Did you feel that knowing others with celiac disease or chronic illness motivated you to adhere to a gluten-free diet?	◯ Yes<br>◯ No
Why not?	______________________________
What factors have helped in adhering to a gluten-free diet?	◯ Family Support<br>◯ Peer Support<br>◯ Mental Health Resources (counseling, therapy)<br>◯ Being involved in schoolwork<br>◯ Being involved in sports, arts, or other extracirriculer activities<br>◯ Other
Other factor	______________________________

What factors have created problems in adhering to a gluten-free diet?	◯ Lack of Family Support<br>◯ Lack of Peer Support<br>◯ Stress from schoolwork<br>◯ Stress from sports, arts, or other extracirriculer activities<br>◯ Other
Other factor	______________________________

Gluten Free Diet Adherence

During the past 30 days, how often have you eaten a food item that contained gluten?	◯ Never<br>◯ 1 time total<br>◯ 2-3 times total<br>◯ 1-2 times per week<br>◯ More than 2 times a week<br>◯ Daily
During the past 30 days, how often have you been careful in reviewing your diet in order to avoid obvious gluten sources?	◯ Never<br>◯ 1 time total<br>◯ 2-3 times total<br>◯ 1-2 times per week<br>◯ More than 2 times a week<br>◯ Daily
During the past 30 days, how often have you been careful in checking medications, supplements, body care products (ie: items that enter nose, eyes, and mouth)?	◯ Never<br>◯ 1 time total<br>◯ 2-3 times total<br>◯ 1-2 times per week<br>◯ More than 2 times a week<br>◯ Daily

Physical Health

Would you say that in general your health is:	◯ Excellent<br>◯ Very Good<br>◯ Good<br>◯ Fair<br>◯ Poor
During the past 30 days, for about how many days have you felt very healthy?	◯ Never<br>◯ 1 time total<br>◯ 2-3 times total<br>◯ 1-2 times per week<br>◯ More than 2 times a week<br>◯ Daily
During the past 30 days, for about how often did poor physical health keep you from doing your usual activities, such as self-care, work, or recreation?	◯ Never<br>◯ 1 time total<br>◯ 2-3 times total<br>◯ 1-2 times per week<br>◯ More than 2 times a week<br>◯ Daily

During the past 30 days, for about how many days did pain make it hard for you to do your usual activities, such as self-care, work, or recreation?	◯ Never<br>◯ 1 time total<br>◯ 2-3 times total<br>◯ 1-2 times per week<br>◯ More than 2 times a week<br>◯ Daily
How frequently do you have doctors visits or use of university health services for celiac disease?	◯ Never<br>◯ Sometimes<br>◯ Often<br>◯ Almost Always

Mental Health

How would you describe your mood?	◯ Excellent<br>◯ Very Good<br>◯ Good<br>◯ Fair<br>◯ Poor
Have you ever felt lonely since entering college or the professional field?	◯ Yes<br>◯ No
Have you had to take any psychotropic medications since entering college or the professional field?	◯ Yes<br>◯ No
Have you had to engage in psychotherapy or other mental health services while in college or when working?	◯ Yes<br>◯ No
How satisfied are you with how this part of your life is going?	◯ Very satisfied<br>◯ Somewhat Satisfied<br>◯ Not satisfied
How much control do you have over this part of your life?	◯ A lot of control<br>◯ Some control<br>◯ No control
How much does celiac disease and/or being on a gluten-free diet impact your ability to function as you would like to during this part of your life?	◯ A significant amount<br>◯ Some amount<br>◯ No amount
Please put your mailing address in this box so we can send you a gift card as a thank you for completing the survey.	______________________________
